# The Focal Play Therapy: A Clinical Approach to Promote Child Health and Family Well-being

**DOI:** 10.3389/fpubh.2019.00077

**Published:** 2019-04-05

**Authors:** Ilaria Chirico, Federica Andrei, Paola Salvatori, Irene Malaguti, Elena Trombini

**Affiliations:** Department of Psychology, University of Bologna, Bologna, Italy

**Keywords:** child eating disorders, child evacuation disorders, parent-child interventions, therapeutic alliance, focal-play therapy

## Abstract

Eating and evacuation disorders can cause serious health problems for children. Early recognition and early treatment require a multifactor intervention based on a collaborative relationship between pediatricians, psychotherapists and other health professionals. In this context the Focal Play Therapy (FPT) with children and parents is a psychodynamic model of intervention that improves parental ability to cope with child's difficulties. Parental engagement in child interventions allows to understand child's symptoms within family dynamics and to build an alliance with parents that represents a crucial variable of an effective psychological support for children and families. In the present study data were collected from 17 parental couples and their preschool children at two time points (1st and 6th FPT sessions) marking the first phase of this intervention. This phase was aimed to the assessment of child's symptoms within family relationships and to the promotion of the alliance with parents. Families were in treatment at the Psychological Consultation Center for Children and Parents located at the Department of Psychology of the University of Bologna (Italy). This Center provides health assessment and intervention services to children and their families. We investigated the alliance from both parents and therapist points of view along with other parental and child outcome variables implied in clinical works with children and families. Alliance scores were obtained through the Working Alliance Inventory and the System for Observing Family Therapy Alliances, two measures used in individual and family settings, respectively. Parenting stress and parent-child interactions were investigated using the Parenting Stress Index and the Emotional Availability Scales. Furthermore, paired *t*-tests were run to detect changes on parental and child variables. Findings advise that special attention should be paid to the building of an early alliance with parents. In this regard the FPT is specifically designed to promote the parent-therapist alliance in the context of child health and family well-being.

## Introduction

The “Focal Play Therapy with children and parents” is a psychodynamic model of intervention specifically designed for child's eating and evacuation disorders in which parents are actively involved in play and a special emphasis is given to the early building of the parent-therapist alliance. Effective interventions for children are needed since eating and evacuation disorders currently represent one of the most frequent reasons of referral to pediatric and infant mental health clinics (1, 2). Common eating disturbances in children are: lower intakes of food than expected for their age, lack of appetite/food-searching behaviors, difficulty with fluids or with foodstuffs, and reluctance or refusal to eat (2, 3). Child eating maladaptive behaviors also include: child's regulation difficulties during feedings, eating only in fixed conditions, and/or being an extremely picky eater (4). Prevalence rates in Western countries range from 25 to 40% of infants referred for under-eating problems (3, 5).

Evacuation disorders consist of constipation, encopresis and soiling and they usually arise in the period of toilet training. Constipation is characterized by low frequency bowel movements leading to encopresis and/or soiling, unusually large amounts of stool, restrictive posturing, and frequently painful voiding (6–9). The world-wide prevalence of child constipation ranges from 0.7 to 29.6% (10), while encopresis has a reported prevalence of 1.5–9.8% in children (11). Without early treatments eating and evacuation disorders tend to persist into adulthood with serious physical damage and medical problems (12, 13).

Child's eating and evacuation disorders put into question the quality of the parent–child relationship. Preschool children usually strive to establish a direct and autonomous relationship with food and corporal contents (14–18). If parents do not facilitate the child's acquisition of autonomy, parent-child relationship problems may occur and they are usually expressed through difficulties concerning eating and evacuation behaviors in children. In these clinical populations, parents who find it difficult to adapt themselves to the child's emerging needs can experience high levels of stress and of psychological impairment. They often have feelings of self-blame and worries about the child's future, isolation, and lack of pleasure activities thus compromising their ability to act constructively in child treatment (19–22).

Nowadays most child-focused interventions involve parents who are responsible for several aspects of the therapeutic process. In this context, clinicians have to build a relationship with parents based on a mutual understanding of the child's problems and on their collaboration/agreement about goals and tasks of child therapy (23–25). These aspects refer to the therapeutic alliance with parents that is a crucial component of a successful child assessment and intervention process (26, 27). A high parent-therapist alliance correlates with low drop-out rates, a decreased youth symptomatology, and improved parenting practices and family functioning (28–30).

Theoretical (problem type, child age, parent sex) and methodological (alliance and outcome reported by the same informant, source and timing of alliance, and outcome assessment) factors may influence the parent-therapist alliance and therapeutic outcome association. As reported by a recent review (31), this association was stronger when the alliance was measured later in treatment and studies evaluated treatment engagement instead of clinical outcomes. Moreover, as expected, weaker correlations were found when the alliance and outcome measures were reported by different informants. For what concerns the remaining factors (problem type, child age, parent sex, source of alliance measurement, timing of outcome measurement), contradictory findings underline the need for future research to understand the specific conditions in which the parent-therapist alliance can predict clinical outcomes and treatment engagement.

Current studies on alliance mainly involve schoolchildren and, simultaneously, their parents attending separate therapy sessions. To our knowledge there is a paucity of data about the assessment of the parent-therapist alliance in the context of preschool psychological treatments involving young children and their families. This gap needs to be filled by new research on different types of child and family treatments.

### The Focal Play Therapy

The “Focal Play Therapy with children and parents” [FPT-CP; (14, 16, 17, 32–34)] consists of weekly alternate play sessions with the child and his/her parents together, and sessions with parents only. As already mentioned, preschool children put effort in establishing a direct and autonomous relationship with food and corporal contents (14–18). When the child's motivation to “do by himself” is coherent with the feeling of being “I” but also part of the family/“We,” the child feels his needs as coherent with parental behaviors and expectations. Otherwise, when parents interfere with the child's acquisition of autonomy, children often express a psychosomatic protest aimed to gain or regain their lost autonomy.

The FPT-CP consists of the therapist's proposal to the child of a temporal sequence during which the main character is a plasticine puppet guided by the therapist. This puppet performs the human basic physiological functions (eating, evacuation, and sleeping) that play a crucial role in the preschool period. The therapist gives voice to the puppet and let it talk about and ask for foods that are prepared with the same materials (plasticine). The puppet seems to appreciate food and, after eating, it expresses the need to urinate and defecate in a potty or toilet bowl built with plasticine. Usually, this sequence is followed by exclamations of relief and comfort.

After this preparatory phase, the therapist allows the child to express through play his psychological contents, desires, fears and internal conflicts and to start managing autonomously the relationship with both food and corporal contents. The FPT-CP main objectives are: re-establishing the natural valence of food and corporal contents and allowing child's direct contact with them through the food selection and preparation, the decision of eating, followed by the need to evacuate and the desire to do it in an appropriate place for family.

During play sessions parents can show two opposite attitudes. On the one hand, they can follow child's creativity in play thus showing patience, collaboration, support and enthusiasm. On the other hand, as for child's eating and evacuation disorders, parents interfere with child's desire of autonomy through impositions, irrelevant or distracting interventions, lack of interest and self-exclusion. In these cases, the clinical work aims to help parents to change their intrusive behaviors toward the child into more adaptive ones. In this regard, clinical evidence has shown that the FPT-CP allows to re-evaluate parental abilities to cope with child's difficulties, to reduce parenting stress and to restore family harmony and well-being (17, 32, 34).

Specifically, the FPT-CP first phase (6 sessions) is aimed to understand the child's symptoms within family dynamics and to promote and maintain a strong parent-therapist alliance as strictly associated to child outcomes. Usually, at the end of this phase, once the therapist has established a positive relationship with the child and his/her parents, the therapist comes to an agreement with both of them about the opportunity to move forward with the therapeutic process in order to obtain a remission of child symptoms. Modalities can be slightly different according to each clinical situation and child/family needs although the FPT-CP structure tends to be the same (i.e., alternate play sessions with the child and his/her parents together and sessions with parents only).

### The Present Study

As previously described, the FPT-CP was specifically designed for children's eating and evacuation disorders and a special emphasis is given to the early building of the parent-therapist alliance as a pre-condition for a successful intervention. To our knowledge, there are only very few data available on the assessment of the parent-therapist alliance in the context of preschool psychological treatments involving young children and their families. This gap needs to be filled by new research on different types of treatment to better inform practice and to improve quality of care for children and their families.

In light of the above-discussed issues, the present study aims to address the quality of the parent-therapist alliance during the first phase (i.e., from session one to session six) of the FPT-CP by means of a multi-method approach. Data were triangulated, namely, therapeutic alliance was investigated from both parents' and therapist's perspectives, and collected longitudinally. Furthermore, in order to assess for congruity, differences in alliance scores among mothers, fathers and therapists were investigated. The present study investigates also the effects of the first FPT-CP phase in terms of reducing parental levels of stress and improving the quality of adult-child relationships.

We hypothesized that: (1) a positive parent-therapist alliance would be developed and maintained throughout the first FPT-CP 6 sessions; (2) there would be a slight initial decrease in the parental levels of stress and a small increase in the quality of adult-child relationships. Regarding the second hypothesis, although triadic interactions represent a unique source of information as they integrate qualities of all family subsystems, they were not evaluated in the present study. This methodological choice was driven by specific theoretical issues concerning the FPT-CP. Indeed, it is a psychodynamic psychotherapy for the child and it does not represent a family therapy or a therapy for parents. Furthermore, because of the nature and severity of child symptoms, we did not hypothesize a high increase in the quality of adult-child relationships. Indeed, as documented in the literature (14, 16, 17, 32–34), in most clinical cases significant changes did not occur in 6 sessions and, therefore, more sessions were required.

## Materials and Methods

### Participants

Families were recruited consecutively from November 2015 to June 2017 at the Psychological Consultation Center for Children and Parents located at the Department of Psychology of the University of Bologna (Italy). This Center provides health assessment and intervention services to children and their families. Parental access to the Center was voluntary.

Participants were 17 couples (*N* = 34; 17 mothers and 17 fathers) and their preschool children (*N* = 17; 13 boys and 4 girls) at their first access to the Center for their child's eating (e.g., food refusal and selective feeding) or evacuation (i.e., constipation and encopresis) problems. Exclusion criteria for the access to the treatment were: (a) child's organic diseases, (b) child's neurodevelopmental disorders, (c) parental past or present psychiatric disorders, (d) parent's lack of proficiency in the Italian language. No exclusion criterion was met by any of the families who took part in the study.

Families were seen by five women psychoanalytic psychotherapists with expertise in the use of the FPT-CP technique. The average patient caseload was approximately three families which were met each once a week.

### Instruments

#### Demographics

An *ad-hoc* questionnaire was created to collect infant information and parental socio-demographic characteristics (e.g., age, nationality, marital status, occupation, and level of education).

#### Therapeutic Alliance

Therapeutic alliance was assessed by means of two measures: the Working Alliance Inventory-Short Form [WAI-SF; (35, 36)] and the System for Observing Family Therapy Alliances-Self report [SOFTA-S; (37, 38)]. The WAI-SF is one of the most used and validated measures of alliance in individual psychotherapy settings. It consists of 12 items and 3 scales: Goal (e.g., “The therapist and I are working toward mutually agreed upon goals”), Task (e.g., “The therapist and I agree about the things I will need to do in therapy to help improve my situation”), and Bond (e.g., “The therapist and I trust one another”). Each item is rated on a 7-point Likert scale (1 = never, 7 = always). The score range of each subscale goes from 4 to 28, whereas the global score ranges from 12 to 84; higher scores reflect more positive ratings of alliance. The reliability and validity of the WAI-SF have been supported in a wide range of studies (39). In the present research Cronbach's (40) alpha total score coefficient was from good (= 0.86) to excellent (= 0.96).

The System for Observing Family Therapy Alliances-Self Report [SOFTA-S; (37, 38)] has been specifically designed to measure the alliance in conjoint settings where the shared sense of purpose within family is essential for positive therapeutic outcomes. It consists of 4 scales: Engagement in the Therapeutic Process (e.g., “The therapist and I work together as a team”), Emotional Connection With the Therapist (e.g., “The therapist has become an important person in my life”), Safety Within the Therapeutic System (e.g., “There are some topics I am afraid to discuss in therapy”), and Shared Sense of Purpose Within the Family (e.g., “Each of us in the family helps the others to get what they want out of therapy”). Clients respond to 16 items on a 5-point Likert scale (1 = not at all, 5 = very much). Each subscale ranges from 4 to 20, while the global score goes from 16 to 80. Higher ratings reflect more positive alliances. In line with previous literature (41, 42) in the present study alpha total score coefficient was from good (= 0.85) to excellent (= 0.92).

Parenting stress. The Parenting Stress Index-Short Form [PSI-SF; (43, 44)] was used to assess parenting stress. It consists of 3 scales: Parental Distress (e.g., “I feel trapped by my responsibilities as a parent”), Parent-Child Dysfunctional Interaction (e.g., “My child rarely does things for me that make me feel good”), and Difficult Child (e.g., “My child seems to cry or fuss more than most children”). Clients respond to 36 items on a 5-point Likert scale (1 = strongly disagree to 5 = strongly agree). Each subscale ranges from 12 to 60, while the global score goes from 36 to 180. In the present study percentile rank classes were used as well. According to the manual (44) scores between the 15th and 84th percentiles are within the normal range for stress; scores between the 85th and 89th percentiles represent a high level of stress and scores ≥90th percentile indicate clinically significant or severe parenting stress. As for the present research, alpha total score coefficient was from acceptable (= 0.78) to excellent (= 0.92).

#### Parent-Child Interactions

Interactions between parents and their children were coded according to the 4th edition of the Infancy/Early Childhood Version of the Emotional Availability Scales [EAS; (45)]. The construct of emotional availability (EA) refers to the parent-child dyad's capacity of a genuine emotional connection (45, 46). The EAS consists of 4 adult scales (Sensitivity, Structuring, Non-Intrusiveness, and Non-Hostility) and 2 child dimensions (Responsiveness and Involvement). Each scale is composed of 7 items and provides a total score computed by summing up scores obtained at each item. Furthermore, a direct score is assigned for each dimension on a 1–7 points Likert scale. Direct scores were used in the present study as they are common for research purposes thus giving an immediate indication of the level of emotional availability displayed by the dyad (45, 46). Two blind raters who were previously trained to reliability in the use of the EAS coded all videos. The degree of agreement between the two coders was measured on a random selection of 30% of the videos. Intraclass correlation coefficient between the two coders was found to be good for research purposes and ranged between 0.68 and 0.85 (mean = 0.79). Following, the EAS dimensions are thoroughly described.

#### Adult Sensitivity

It evaluates the adult's appropriate and positive affective exchanges consisting of an adequate perception of emotions, responsiveness to the child's cues, ability to handle conflictual situations, and awareness of timing. High-end scores (6–7) represent optimal sensitivity, the mid-point ratings (4–5) refer to inconsistent/apparent sensitivity, and the lowest scores (1-2-3) represent emotional detachment.

#### Adult Structuring

It refers to the parent's ability to guide the child during play. High-end scores indicate optimal structuring, the mid-point ratings indicate inconsistent structuring (mismatch between the adult and the child, i.e., there may be too much structuring in a way that the child cannot absorb it), and the lowest scores represent a lack of adult's structuring in the interactions.

#### Adult Non-intrusiveness

It investigates the absence of over-directions, overstimulation, interferences or over-protection in the adult's behavior. High-end scores indicate that the adult is a non-intrusive and a supportive presence, middle-range scores represent benign intrusiveness and over protectiveness, low-end ratings indicate adult intrusiveness, and physical intrusion.

#### Adult Non-hostility

It evaluates the absence of adult hostile behaviors (covert or overt) toward the child. Hostile behaviors include verbal or physical aggressiveness (overt), and the adult's subtle expressions of boredom, impatience, frustration (covert). High-end scores indicate a lack of any hostility in face, voice or bodily actions; middle range ratings indicate covert hostility; and lower scores indicate overt hostility.

#### Child's Responsiveness

It measures the quality of the child's affect and responsiveness to the adult. High-end scores refer to a child who is emotionally connected to the adult in an age-appropriate way. Middle range scores indicate a child who is connected but he/she tends to be over solicitous to the adult's directions with limits on child's autonomy. Low-end ratings indicate an either over-connected or under-connected child who may/or may not reflect a disorganized-traumatized affective relationship with the caregiver.

#### Child's Involvement

It refers to the child's capacity to engage the adult in the interaction. High-end scores indicate the child's ability and interest in taking the initiative in the interaction. Middle-point ratings reflect the child's way to engage the adult that is characterized by negative emotions, distress or crisis scenarios. Low-range scores indicate the child's passivity or lack of interest in the relationship with the adult.

### Procedure

The research was approved by the Ethic Committee of the University of Bologna (Italy). Participation in the present study was based on the family's informed and signed consent. Informed consent included confidentiality and the client's right to withdraw at any time. Families were screened in terms of the previously mentioned exclusion criteria and they were assigned to therapists according to availability.

The present research focused on a specific phase of this intervention represented by the first 6 sessions aimed to the assessment of child symptoms and to the promotion of the parent-therapist alliance. This therapeutic relationship consists of a mutual understanding of child difficulties and, also, of the parental collaboration and agreement on main goals and tasks of the intervention. In this sense the parent-therapist alliance is a prerequisite to treatment integrity. Below are the 7 FPT-CP sessions where data collection occurred:

- 1st session: with parents;
- 2nd session: with the child and his/her parents;
- 3rd session: with the child and his/her mother;
- 4th session: with parents;
- 5th session: with the child and his/her father;
- 6th session: with parents;
- 7th session: with the child and his/her parents.

During sessions with parents, the therapist focuses on child's difficulties, themes and family topics emerged in play sessions with the child. As explained above, the FPT-CP main purposes are: re-establishing the natural valence of food and corporal contents for children and allowing their direct contact with them (14, 16, 17, 32–34). During play sessions parents can show two opposite attitudes. In particular, positive parental behaviors are characterized by tolerance, patience, collaboration, support and proposals in line with child's creativity, along with a trust and enthusiasm in his play abilities. By contrast, as for child's eating and evacuation disorders, parental behaviors consist of impositions rather than proposals, irrelevant or distracting interventions, lack of interest and self-exclusion. These parental behaviors are thoroughly discussed during sessions with parents in order to allow a shift from parental intrusive and coercive behaviors toward the child into more adaptive ones.

As previously mentioned, the FPT-CP is a psychodynamic psychotherapy for children and it does not represent a family therapy or a therapy for parents. Indeed, in cases in which high marital conflicts had a negative impact on child-parent and co-parenting relationships, they were taken into account and thoroughly discussed by the therapist in the FPT-CP first phase. However, as it is a child-focused intervention, a couple therapy and/or more appropriate interventions for family needs were recommended.

An *ad-hoc* socio-demographic questionnaire was given to families before treatment. At the end of the 1st and 6th sessions (marking the FPT-CP first phase) parents completed self-reports about the therapeutic alliance and parenting stress. Parallel alliance measures were completed by therapists as well.

In order to assess changes in the quality of adult-child relationships, dyadic interactions were evaluated at the beginning of the 2nd (before treatment) and during the 7th (where only data collection occurred without treatment) sessions. Assessments took place during two consecutive 10-min sessions video recorded continuously by a female filmer at the Psychological Consultation Center for Children and Parents (Department of Psychology, University of Bologna). In a quiet room parents were asked to play individually with their child in ways they typically would and to disregard the filmer's presence as much as possible. A set of standard, age-appropriate toys was used and families were allowed to use any toys and puppets provided.

### Statistical Analysis

Data analysis was performed using IBM SPSS version 20.0 for Windows. A *p* < 0.05 indicated statistical significance.

The first section of Results provide descriptive statistics for each WAI-SF and SOFTA-S scale for mothers, fathers and therapists at two time points (T1 and T2). Student's *t*-test was used to examine differences between males and females and, moreover, to compare each parent and therapist alliance scores. For what concerns the SOFTA-S—that measures the alliance perceived by the therapist with the family as a “unit”—firstly a family score was calculated for each scale (the average ratings of mothers and fathers). Secondly, unpaired *t*-tests were conducted to analyze differences between the therapist and the family alliance scores. The following sections present group comparisons between mothers and fathers in terms of parent PSI-SF and parent and child EAS mean scores.

## Results

Ages of mothers and fathers ranged from 34 to 53 years (*M* = 41.41, *SD* = 5.04) and 32 to 48 years (*M* = 42.41, *SD* = 4.76), respectively. Children aged 2–5 (*M* = 3.87, *SD* = 1.43) were referred for evacuation (60%) or eating (40%) problems. Parents were Italian, married (88.2%) or cohabiting, and all of them were employed. With regards to educational level, most mothers had a university degree (82.3%), and a few completed only secondary (11.8%) or middle school (5.9%). Like the mothers, most of the fathers obtained an academic degree (64.7%), while smaller percentages finished secondary (11.8%) or middle school (23.5%).

### Alliance

Table 1 presents descriptive statistics for the WAI-SF and SOFTA-S total scores at the 1st (T1) and 6th (T2) sessions. Parent and therapist alliance scores were high and indicative of a positive alliance at each time of assessment (27, 38). Moreover, paired *t*-tests did not reveal significant differences between T1 and T2 thus confirming the development and maintenance of a good parent-therapist alliance throughout the first FPT-CP 6 sessions.

**Table 1:** Means, SDs ofWAI-SF and SOFTA-S at T1 and T2, and tests for differences.

	**T1**	**T2**	***t*_**(16)**_**	***p***	***d***
	**M ± SD**	**M ± SD**			
**MATERNAL SCALES**					
WAI-SF total	70.71 ± 8.61	70.53 ± 8.24	0.07	0.94	0.02
SOFTA-S total	68.12 ± 7.79	67.29 ± 7.77	0.43	0.67	0.11
**PATERNAL SCALES**					
WAI-SF total	70.47 ± 11.09	68.00 ± 8.75	0.90	0.38	0.25
SOFTA-S total	68.06 ± 7.04	68.12 ± 5.97	−0.04	0.97	0.01
**THERAPIST SCALES**					
WAI-SF t-m total	59.12 ± 8.74	60.06 ± 11.03	−0.38	0.71	0.09
WAI-SF t-f total	54.00 ± 11.41	55.88 ± 11.71	−0.75	0.46	0.16
SOFTA-S t-family total	55.76 ± 9.24	57.65 ± 9.91	−0.89	0.39	0.20

*t-m, therapist's alliance with mother; t-f, therapist's alliance with father*.

While parents did not significantly differ in the WAI-SF scores, at T1 the therapist ratings of alliance with mothers were significantly higher than those ones with fathers [WAI-SF Total: M_therapist−mother_ = 59.12 vs. M_therapist−father_ = 54.00; *t*_(16)_ = 2.57, *p* = 0.02]. Differences did not emerge at T2.

For what concerns the SOFTA-S, the therapist scores were significantly lower than the family ratings of alliance at both time points [SOFTA-S Total: T1 M_therapist_ = 55.76 vs. M_family_ = 68.09, *t*_(32)_ = 4.47, *p* = 0.00; T2 M_therapist_ = 57.65 vs. M_family_ = 67.71, *t*_(32)_ = 3.54, *p* = 0.00].

### Parenting Stress

Descriptive statistics for the PSI-SF are reported in Table 2. No differences emerged between T1 and T2, although fathers showed a trend with increased stress scores—from session one to session six—that did not reach statistical significance.

**Table 2:** Means, SDs of PSI-SF at T1 and T2, and tests for differences.

	**T1**	**T2**	***t*_(16)_**	***p***	***d***
	**M ± SD**	**M ± SD**			
**MATERNAL SCALES**					
Parental distress	28.88 ± 9.02	29.18 ± 8.45	−0.24	0.81	0.03
Parent-child dysf unctional interaction	23.12 ± 5.80	22.12 ± 5.69	0.82	0.42	0.17
Difficult child	31.94 ± 8.59	30.94 ± 8.09	0.72	0.48	0.12
Total	83.94 ± 18.77	82.24 ± 18.10	0.54	0.60	0.09
**PATERNAL SCALES**					
Parental distress	27.41 ± 6.57	28.71 ± 6.89	−0.95	0.36	0.19
Parent-child dysfunctional interaction	23.71 ± 6.53	25.06 ± 6.15	−0.99	0.34	0.21
Difficult child	31.65 ± 6.90	33.29 ± 6.18	−0.85	0.41	0.25
Total	82.76 ± 14.18	87.06 ± 13.16	−1.35	0.20	0.31

According to the PSI-SF manual (44) mothers reported high levels of stress on the Difficult Child Scale (T1: 90th percentile, T2: 85th percentile), while fathers obtained clinically significant scores on the Difficult Child scale (T1: 85th percentile, T2: 90th percentile) and on the Total score as well (T1: 80th percentile—still not clinically relevant, T2: 85th percentile).

### Parent-Child Interactions

Table 3 reports mean scores on the parental and child dimensions of the EAS. No statistically significant differences emerged between T1 and T2.

**Table 3:** Means, SDs of EAS at T1 and T2, and tests for differences.

	**T1**	**T2**	***t*_(16)_**	***p***	***d***
	**M ± SD**	**M ± SD**			
**MOTHER-CHILD SCALES**					
Sensitivity	5.97 ± 1.12	5.91 ± 1.19	0.33	0.74	0.05
Structuring	4.94 ± 1.33	4.65 ± 1.28	1.13	0.28	0.22
Non-Intrusiveness	6.32 ± 0.87	6.03 ± 1.18	1.21	0.24	0.28
Non-Hostility	6.62 ± 0.49	6.59 ± 0.59	0.44	0.67	0.06
Responsiveness	5.47 ± 1.39	5.59 ± 1.54	−0.32	0.75	0.08
Involvement	4.85 ± 1.30	5.12 ± 1.67	−0.77	0.45	0.18
**FATHER-CHILD SCALES**					
Sensitivity	4.59 ± 1.53	4.88 ± 1.59	−0.77	0.45	0.19
Structuring	3.77 ± 1.39	4.38 ± 1.71	−1.84	0.09	0.39
Non-Intrusiveness	5.94 ± 0.68	5.94 ± 0.68	0.00	1.00	0.00
Non-Hostility	6.56 ± 0.66	6.38 ± 0.86	1.14	0.27	0.23
Responsiveness	4.65 ± 1.61	4.79 ± 1.80	−0.43	0.67	0.08
Involvement	3.79 ± 1.68	4.00 ± 1.94	−0.82	0.42	0.12

According to the EAS manual (45), mothers reported problems on the dimension of Structuring (T1: *M* = 4.94, T2: *M* = 4.65), and children on the Involvement scale (T1: *M* = 4.85, T2: *M* = 5.12). With regards to fathers in the present sample, they reported problematic scores on the two dimensions of Sensitivity (T1: *M* = 4.59, T2: *M* = 4.88) and Structuring (T1: *M* = 3.77, T2: *M* = 4.38), and children on the Responsiveness (T1: *M* = 4.65, T2: *M* = 4.79) and Involvement (T1: *M* = 3.79, T2: *M* = 4.00) scales.

## Discussion

Attention to the parental engagement in child treatment has recently increased given the emphasis on implementing successful treatments into community settings, identifying methods to provide services more efficiently, and improving quality of care for children and families (47–49). The study described herein focused on the FPT-CP as a child-focused psychotherapeutic technique designed for eating and evacuation disorders during preschool years. As discussed above, main purposes of this intervention are re-establishing the natural valence of food and corporal contents for children while supporting adults to re-evaluate their parental abilities. Our first goal was to explore the quality of the parent-therapist relationship during the first FPT-CP 6 sessions aimed to the promotion of the therapeutic alliance with parents and to the assessment of child's symptoms within family dynamics. We investigated also the potential effects of these initial sessions in reducing parental levels of stress and improving the quality of adult-child relationships.

Regarding our first hypothesis, results confirmed our expectations. High levels of parent-therapist alliance were promoted and maintained throughout the FPT-CP first phase. Parents were highly motivated and in need of help for their child's problems and, since the beginning of the intervention, they trusted in the therapist's ability to help them. At the same time the intra-family collaboration was consistently promoted by therapists who worked to build a positive family emotional climate that was necessary for the child and family disclosure throughout sessions. In this regard the use of the SOFTA-S allowed to evaluate some specific characteristics of a conjoint psychotherapeutic setting (with more than one family member) where the family productive collaboration and shared sense of purpose are strictly associated to therapeutic outcomes.

In the present study gender differences were taken into account. As expected, while we did not find differences between mother and father alliance scores, parental ratings of alliance were significantly higher than the therapist scores. These results were not surprising since the client and therapist views of alliance can diverge (50, 51). Therapist's perceptions of alliance might be affected by the theoretical knowledge and, most importantly, clients in the present sample were highly motivated and the family access to the Service was voluntary.

Instead unexpected results were obtained when comparing the therapist's ratings of alliance with mothers and fathers. Interestingly we found that, at the end of the FPT-CP 1st session, the therapist-mother alliance scores were significantly higher than the therapist-father ratings of alliance. Since the therapist sample consisted of women only, possible explanations of these results may come from the social psychology studies according to which people of the same sex tend to view the world through the same gender lens, which in turn might lead to similar life-perspectives (52). What is relevant herein is that, at the end of the FPT-CP 6th session, there were no differences between therapist-mother and therapist-father alliance scores. In other words, it seems that the building of the therapeutic relationship with fathers—as much positive as with mothers—was not immediate but it occurred throughout the first 6 sessions (by the therapist side).

Nowadays very little information is available about the effects of involving fathers in child treatment. Despite many aspects have been changed in the distribution of parental responsibilities, mothers are often exclusive participants in the early child intervention service delivery (53). Indeed to a certain extent there is still the belief that fathers have a limited role in childcare, or that they are difficult to recruit in child treatment (54, 55). Hence findings from the present research shed light on the importance of new research on those therapist factors that might contribute to the psychotherapy process and outcome. Among them are the therapist gender, expectations, stereotypes, and internal attitudes that are shaped and re-shaped over the course of treatment (56).

As previously discussed, without early diagnosis and effective child and family treatments, eating and evacuation disorders can cause serious child medical problems and high levels of parental maladjustment to cope with child difficulties. In line with our second hypothesis, we found high levels of parental distress on the Difficult Child scale that measures how much parents perceive the child as difficult or easy to manage (43). Unexpectedly, although scores did not significantly decrease from the 1st to the 6th sessions, at a qualitative level mothers and fathers reported different patterns of stress development. While maternal perceptions of the child as “difficult” started to change toward a deeper understanding of parent-child difficulties, father stress scores slightly increased on the Difficult Child and Total scales. Most probably, at the end of the FPT-CP first phase fathers were more involved in family life and they achieved a greater awareness about the existing problems thus leading to somewhat higher levels of distress.

Regarding our third goal, we found that mothers and fathers reported problems on the Structuring scale that refers to the adult capacity to appropriately facilitate, scaffold, or organize the child play. Specifically they showed over-structuring or attempts to structure that were not well-received by the child and that, at the end, were unsuccessful. These results are consistent with the etiology of eating and evacuation disorders during the preschool years where child's emerging needs of autonomy can conflict with family. It is interesting to note that fathers only reported low levels of emotional availability showing a warm and kind parental attitude though not sensitive to child cues and communications. It might be that, compared to mothers, fathers were less used to interact with their children and to interpret their signals and behaviors.

Overall findings from the present research show that the first FPT-CP 6 sessions were effective in promoting a positive parent-therapist alliance as a pre-condition for a successful child and family treatment. However, changes in parental levels of distress and parent-child relationships did not reach statistical significance and we can speculate that more sessions were needed to obtain a remission of child symptoms.

Some limitations of the study must be considered when interpreting our results. Firstly, due to the small sample size, findings should be replicated on larger samples and results should be interpreted with caution. Secondly, the use of the alliance questionnaires did not allow to capture specific client and therapist behaviors that shape the alliance during treatment. It would be interesting to explore through further longitudinal studies how the pattern of alliance, stress and adult-child interactions observed herein would evolve in a longitudinal way. Moreover, future research would benefit from investigating the parent-therapist alliance in the context of different models of child-focused treatment.

Despite these limitations, findings from the present research would highlight relevant clinical implications. It is well-known that, without early treatments, eating and evacuation disorders tend to persist into adulthood with serious effects on physical and mental health (12, 13). The FPT-CP has been designed for eating and evacuation disorders in preschool children usually connected to parent-child relationship problems. During sessions parents are trained to be more sensitive toward child's needs and they start to re-evaluate their parental abilities in supporting child's emerging needs of autonomy in his relationship with food and corporal contents. This clinical methodology is characterized by the use of play as a narrative and central dimension of child/family problems (24). Furthermore, it is based on a high parental engagement in therapy sessions as precondition for successful clinical outcomes. Therapists with expertise in the use of the FPT-CP are trained to achieve high levels of parental session-engagement through the building of a strong parent-therapist alliance. Parents at risk for poorer alliance are identified and the early intervention is adapted to improve early alliance and to reduce dropouts.

To conclude, for the above mentioned reasons, the FPT-CP is a specific child-focused intervention that might represent a preventive model to apply to clinical contexts both public and private ones. Future research efforts should develop treatments for preschoolers based on parent-professional alliance and aimed to provide psychological support to families and to enhance the parental abilities to cope with child diseases in preschool years.

## Author Contributions

IC and ET contributed to the conception and design of the study. IC acquired, analyzed, and interpreted data. IC and FA drafted and reviewed the initial and final manuscript as submitted. PS and IM collected data and they gave technical support and advice. All authors contributed to the manuscript revision, read, and approved the submitted version.

### Conflict of Interest Statement

The authors declare that the research was conducted in the absence of any commercial or financial relationships that could be construed as a potential conflict of interest.
